# Childbirth-related posttraumatic stress symptoms – examining associations with hair endocannabinoid concentrations during pregnancy and lifetime trauma

**DOI:** 10.1038/s41398-023-02610-3

**Published:** 2023-10-31

**Authors:** Luisa Bergunde, Marlene Karl, Sarah Schälicke, Victoria Weise, Judith T. Mack, Tilmann von Soest, Wei Gao, Kerstin Weidner, Susan Garthus-Niegel, Susann Steudte-Schmiedgen

**Affiliations:** 1 Institute and Policlinic of Occupational and Social Medicine, Faculty of Medicine, Technische Universität Dresden, Dresden, Germany; 2 Department of Psychotherapy and Psychosomatic Medicine, Faculty of Medicine, Technische Universität Dresden, Dresden, Germany; 3 PROMENTA Research Center, Department of Psychology, University of Oslo, Oslo, Norway; 4 Institute of Biological Psychology, Faculty of Psychology, Technische Universität Dresden, Dresden, Germany; 5 Institute for Systems Medicine (ISM), Faculty of Medicine, Medical School Hamburg MSH, Hamburg, Germany; 6 Department of Childhood and Families, Norwegian Institute of Public Health, Oslo, Norway

**Keywords:** Human behaviour, Predictive markers, Psychiatric disorders

## Abstract

Evidence has linked alterations of the endocannabinoid system with trauma exposure and posttraumatic stress disorder (PTSD). Childbirth-related PTSD symptoms (CB-PTSS) affect about every eighth woman and can negatively influence the entire family. While aetiological models of CB-PTSD include psychological risk factors such as maternal trauma history and negative subjective birth experience (SBE), they lack biological risk indicators. We investigated whether lifetime trauma and CB-PTSS were associated with long-term endocannabinoid concentrations during pregnancy. Further, we tested endocannabinoids as mediators between lifetime trauma and CB-PTSS and whether SBE moderated such mediational paths. Within the prospective cohort study DREAM_HAIR_, 263 expectant mothers completed trauma assessments and provided hair samples for quantification of long-term endocannabinoid levels (anandamide [AEA], 2-arachidonoylglycerol [1-AG/2-AG], and N-acyl-ethanolamides [NAE]) prior to their anticipated birth date. Two months postpartum, CB-PTSS and SBE were measured. Regression models controlling for relevant confounders showed no association between lifetime trauma and hair endocannabinoids during pregnancy, yet higher number of lifetime trauma events and lower hair AEA were significantly associated with CB-PTSS, with the latter finding not remaining significant when Bonferroni corrections due to multiple testing were applied. While hair AEA did not mediate the association between lifetime trauma and CB-PTSS, the effect of lower hair AEA on CB-PTSS was stronger upon negative SBE. Results suggest greater lifetime trauma and reduced maternal hair AEA during pregnancy may be associated with increased risk for CB-PTSS, particularly upon negative SBE. Findings confirm lifetime trauma as a CB-PTSS risk factor and add important preliminary insights on the role of endocannabinoid ligand alterations and SBE in CB-PTSS pathology.

## Introduction

While childbirth is widely considered a joyful event, on average 12.3% of women in community samples develop childbirth-related posttraumatic stress disorder (CB-PTSD) symptoms after childbirth [1]. These posttraumatic reactions, including reexperiencing, avoidance, and arousal symptoms, represent an important public health issue [2]. Hence, a better aetiological understanding through extension of psychological models with biological factors is warranted [3].

Previous psychological research has been synthesised in a diathesis-stress model for CB-PTSD [4], detailing how CB-PTSD is influenced by pre-birth risk factors, such as prior traumatic events, perinatal depression, and fear of childbirth (FOC), as well as birth-related risk factors, such as negative subjective birth experience (SBE), and postnatal factors, such as social support. Empirical evidence suggests the incidence and severity of general PTSD may depend on the number of traumatic event types experienced (hereafter termed ‘lifetime trauma’), a mechanism that has been termed the *building block effect* [5, 6]. Lev-Wiesel et al. [7] provided preliminary evidence for this effect regarding CB-PTSD, showing greater lifetime trauma predicted maternal CB-PTSD symptoms (CB-PTSS). Further, research emphasises women’s negative SBE as a key risk factor of CB-PTSS (e.g., [8, 9]), highlighting the importance of subjective peritraumatic experiences for CB-PTSD.

Outside childbirth, biological markers (e.g., molecular, neuroanatomical) of trauma exposure and PTSD have been investigated [10, 11]. PTSD is commonly characterised as a failure of the stress response system to regain homoeostasis [12]. It follows that dysregulations of the hypothalamus-pituitary-adrenal (HPA) axis, our major neuroendocrine stress response system involving glucocorticoid (GC) secretion, have been repeatedly linked to trauma exposure and PTSD development [13, 14]. Far less is known regarding biological markers in CB-PTSD, with our group recently providing initial evidence for the role of long-term GCs as predictors for CB-PTSS upon negative SBE [15]. Besides the HPA axis, the endocannabinoid system (ECS) is gaining attention in trauma- and PTSD-related research through its role in adaptive memory processes, anti-inflammatory processes, and regulation of the stress response [16] and is discussed as a potentially promising pharmacological treatment target [17]. Specifically, available evidence suggests that acute stress and associated GC secretion (e.g., cortisol – the primary HPA axis outcome) can influence endocannabinoid (EC) concentrations, whereby the ECS modulates HPA axis activity, hindering overshooting behavioural stress responses [18]. Hence, latest conceptual models characterise the ECS as a potential stress-buffer, with the tonic activity of the endocannabinoid anandamide (AEA) regulating HPA axis activity and phasic increases in 2-arachidonoylglycerol (2-AG) aiding its termination, thereby functioning to regulate homoeostasis [19, 20]. In addition to the ECs AEA and 2-AG, the ECS consists of receptors CB_1_ and CB_2_, enzymes and transport proteins, and endocannabinoid-related compounds (ERCs), the N-acyl-ethanolamides (NAE) (i.e., palmitoylethanolamide (PEA), oleoylethanolamide (OEA), and stearoylethanolamide (SEA)).

Consistent with conceptual models, animal and human research has linked altered ECS activity with dysregulated stress reactivity and development of trauma-related psychopathology [20–22]. Specifically, animal research indicates that augmentation of AEA signalling through pharmacological blocking of the AEA-metabolising enzyme FAAH enhances extinction learning [23, 24], which plays an important role in PTSD development [25]. In line with this, a randomised controlled experimental medicine trial by Mayo et al. [26] showed that humans who received an FAAH inhibitor also exhibited improved fear extinction memory recall, reduced autonomic stress reactivity, and reduced stress-induced negative affect. Furthermore, in humans the drug rimonabant, which blocks EC receptors in the brain and periphery, has been shown to induce psychiatric side effects including depression and anxiety [27]. Considering the ERCs may exert so-called entourage effects to AEA, potentiating AEA’s effects through activation of other receptors such as the transient receptor potential vanilloid channel (TRPV_1_) [28], and also being metabolised amongst others by FAAH [29], it fits that findings also document reductions in AEA and ERCs after trauma and in PTSD [21]. This is also consistent with their anti-inflammatory, analgesic, and neuroprotective effects [30]. As described above, 2-AG signalling appears to regulate acute termination and long-term habituation of the stress system [16], with preclinical research associating 2-AG depletion with increased anxiety-like behaviours in rats [31] and individuals with PTSD showing reduced circulating 2-AG levels [32]. This underscores the anxiolytic function of 2-AG, however more complex biphasic actions are assumed to be present [17]. Focusing on observational human research, reduced [32, 33] and elevated [34, 35] circulating EC/ERC levels measured in blood have been found in PTSD patients compared to healthy controls, supporting ECS dysregulation in PTSD. In this line of research heterogeneity regarding direction of dysregulation remains, also in longitudinal investigations [36], and may be attributable to measurements in blood being highly influenced by situational factors (reviewed in [37]).

To circumvent these limitations, recently, ECs (i.e., AEA and 2-AG which is typically quantified as the sum of 2-AG and its isomer 1-AG) and ERCs (i.e., the NAEs SEA, PEA, and OEA) have been quantified in human hair, enabling less intrusive sampling, reduced bias from daily variations, higher reliability, and retrospective assessments over several months [38]. Wilker et al. [39] found lower hair EC/ERC concentrations (HEC), specifically in PEA, OEA, and SEA levels, to be associated with greater lifetime trauma and more severe PTSS in a sample of traumatised war survivors. These findings indicate a link between reduced HEC and PTSD aetiology and suggest a dose-response relationship between HEC on the one hand and PTSS severity and lifetime trauma on the other hand. Although cross-sectional, findings align with the psychological building block effect [5] and possible biological explanations of the effect, where a dysregulation of the long-term endocrine stress response system has been suggested to explain this link [14].

Subsequent research supporting higher EC/ERC levels as a stress-buffer and indicating reduced EC/ERC levels as a potential biological mechanism of the building block effect reported a negative association of hair AEA with anxiety [38] and depressive symptoms [40], lower hair AEA in borderline personality disorder patients [41], and found more severe childhood maltreatment associated with lower hair SEA amongst pregnant women in the last trimester [42]. However, other studies found no association between HEC and PTSS or a positive association between traumatic experiences and 2-AG, OEA, and PEA [43, 44]. Taken together, available studies are limited due to small sample sizes and partial inability to measure AEA and 2-AG. Furthermore, although studies examining HEC in the pre- and postnatal period exist [42, 45], further investigation is necessary as the perinatal period shows specific physiological changes and mental illnesses [46, 47].

In this investigation, we therefore aimed to assess (i) the relationship between mothers’ lifetime trauma and HEC estimates during pregnancy, (ii) the association between HEC during pregnancy and subsequent CB-PTSS, and (iii) whether HEC mediate the relationship between lifetime trauma and CB-PTSS. Considering current evidence indicates an overall downregulation of the ECS and EC/ERC ligands after trauma [21] and in relation to PTSD [16, 39] no specific hypotheses are derived for individual ECs/ERCs. By integrating the building block effect of lifetime trauma [5, 14] with conceptual models of ECS as a stress-buffer [20], we expected lifetime trauma to predict reduced HEC [39] and elevated CB-PTSS [7], and reduced HEC during pregnancy to predict CB-PTSS [39]. Further, integrating the ECS into Ayers et al.‘s. [4] model of CB-PTSD, we hypothesised that the relationship between lifetime trauma and CB-PTSS is mediated by HEC [21]. Finally, based on our previous GC findings using a subset of the present sample detecting a predictive relationship only when including SBE [15], we expected SBE to moderate the association of lifetime trauma and HEC with CB-PTSS, such that relationships are stronger for more negative SBE.

## Methods

### Study design and participants

**Fig. 1:**
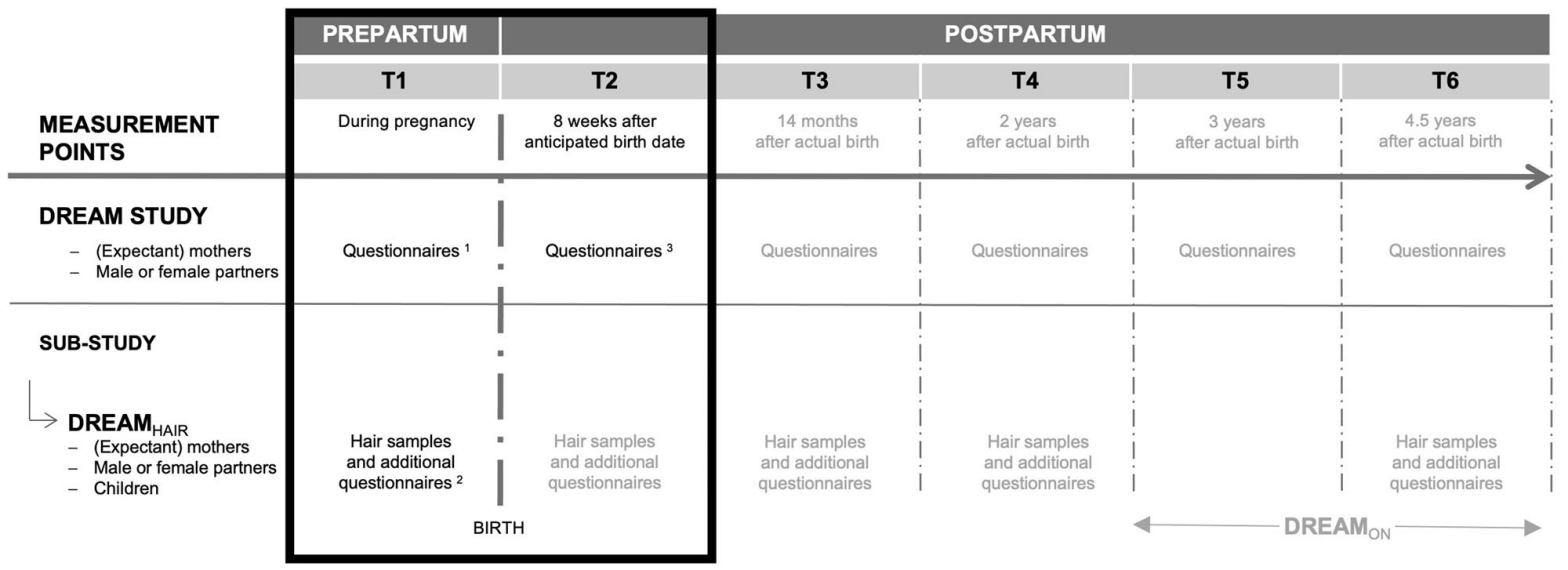
Measurement waves of the DREAM study and its endocrine sub-study DREAM_HAIR_. Greyed assessments are not relevant to this investigation. This study’s sample completed ^1^questionnaires at T1 DREAM (*M* = 26.67 weeks pregnant, *SD* = 5.37 weeks, *Range* = 12–40 weeks pregnant), ^2^completed questionnaires and provided hair samples at T1 DREAM_HAIR_ (*M* = 22.10 days before the actual birth date, *SD* = 10.64 days, *Range* = 1–47 days), and completed ^3^questionnaires at T2 DREAM (*M* = 8.24 weeks, *SD* = 1.55 weeks, *Range* = 4–14 weeks).

This study used data from the prospective cohort study DREAM (Dresden Study on Parenting, Work, and Mental Health) and its endocrine sub-study DREAM_HAIR_ [48]. DREAM_HAIR_ investigates long-term endocrine determinants of perinatal stress and mental health-related outcomes in mothers, partners, and offspring across five assessments (T1 DREAM_HAIR_ – T6 DREAM_HAIR_, Fig. 1).

Expectant parents were recruited (July 2017 – December 2020) first for DREAM mainly at birth information evenings in Dresden, Germany. If T1 DREAM questionnaires were completed at least four weeks before the anticipated birth date, participants were screened for DREAM_HAIR_ via telephone and took hair samples 4 ± 2 weeks prepartum. T2 DREAM questionnaires were sent postally 8 weeks after the anticipated birth date.

**Fig. 2:**
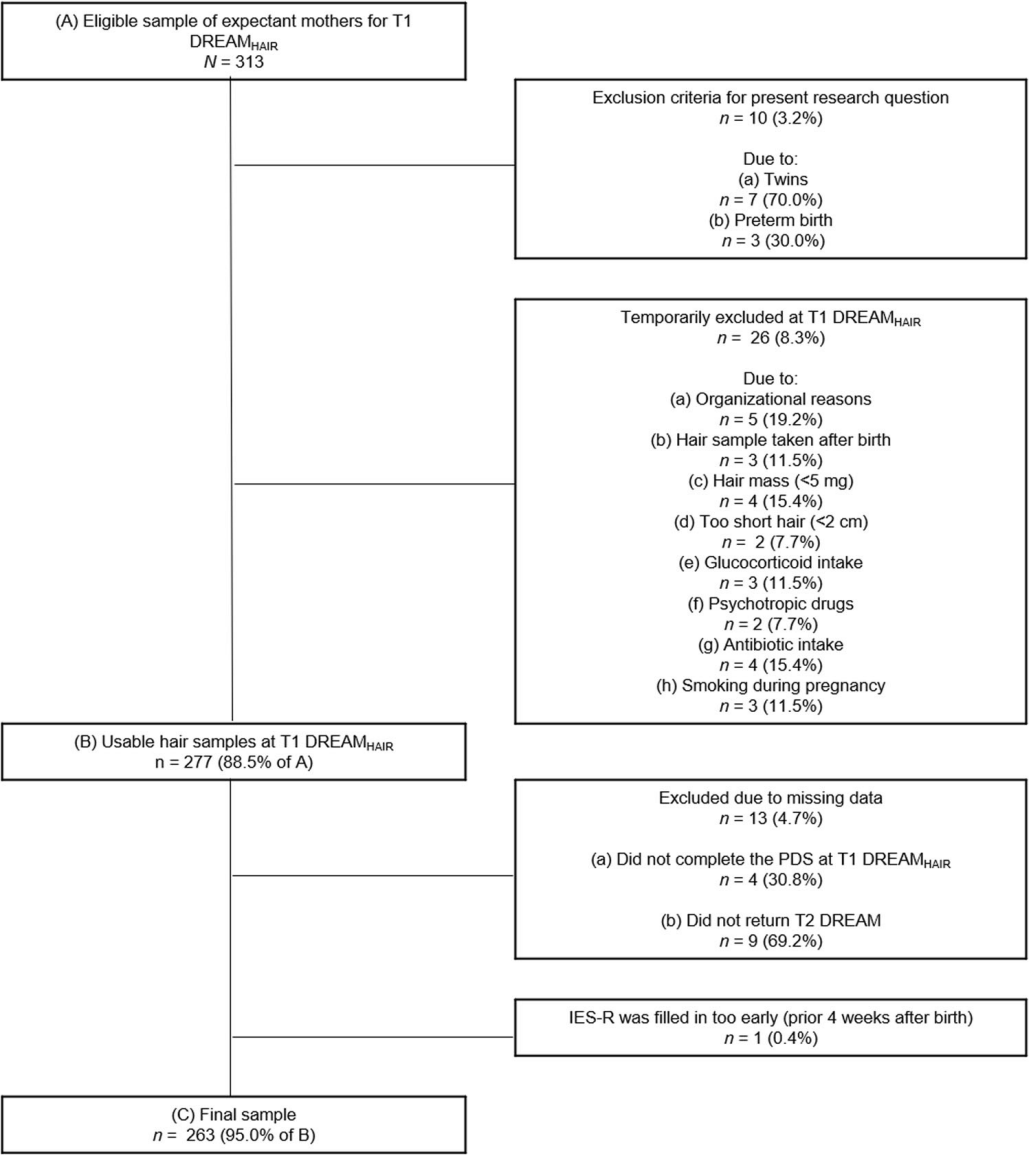
Flowchart of retention rate and exclusion criteria resulting in final sample. Data until January 31st 2022 (version 9 of the quality-assured data files, prospective data collection ongoing). T1 DREAM_HAIR_ = 4 ± 2 weeks prior to anticipated birth date; T2 DREAM = 8 weeks after anticipated birth date. PDS Posttraumatic Diagnostic Scale, IES-R Impact of Event Scale-Revised.

DREAM study inclusion criteria were being currently pregnant, resident in Dresden (Germany), and sufficient German language knowledge. Participation in DREAM_HAIR_ further required minimum 2 cm hair length, no baldness or hair loss, no severe illness over the past five years, and no glucocorticoid-containing medication in the past three months. For this investigation further inclusion criteria were no multiples or preterm births because of a potentially different pregnancy and birth experience and no behaviour likely altering HEC (e.g., smoking; [49]). Wrong timing, length, and mass of hair sampling, failure to complete T1 DREAM_HAIR_ and T2 DREAM, and delayed sending of samples to the laboratory resulted in exclusion. Also, we excluded one participant as she completed T2 DREAM prior four weeks postpartum, whereby her Impact of Event Scale-Revised (IES-R; [50]) score represented acute stress disorder symptoms rather than PTSS [51]. The final sample comprised *N* = 263 expectant mothers (see Fig. 2).

Attrition analyses showed that non-completers (T2 DREAM not completed; *n* = 9) did not differ from completers (participated at T1 DREAM, T1 DREAM_HAIR_, and T2 DREAM; *n* = 264) regarding any study variables (tables on request).

### Measures

#### Sociodemographic and hair characteristics

Sociodemographic characteristics (e.g., age, pre-pregnancy BMI, academic degree), medication intake, and physical illness were assessed at T1 DREAM. An in-house hair protocol at T1 DREAM_HAIR_ [52] assessed self-reported hair-related characteristics (e.g., weekly hair washes, any hair treatment including perm, tint, or colouring), health-related questions (e.g., smoking and alcohol), and medication intake (e.g., psychotropic drugs) regarding the past three months. We further grouped participants regarding exposure to the COVID-19 pandemic (cut-off date March 9th, 2020; [53]) to check for effects on HEC and CB-PTSS.

#### Psychological measures

CB-PTSS was the main outcome variable and assessed at T2 DREAM using the IES-R (German version; [50, 54]). Traumatic stress in relation to childbirth in the past week was assessed with the subscales *intrusion, avoidance*, and *hyperarousal*. Participants rated 22 items on a 4-point scale from *not at all* (0) to *often* (5) (total score: 0–110; [55]). The original IES’s (total score of intrusion and avoidance scales: 0–75) clinical cut-off of ≥35 was used [56].

Lifetime trauma (i.e., sum of number of potentially traumatic event types experienced) was measured by the self-report Trauma Checklist of the Posttraumatic Diagnostic Scale at T1 DREAM_HAIR_ (PDS; [57]). Participants specified which (if any) of 12 traumatic event types (including an open category “other”) they had experienced in their lifetime. All events under “other” that related to childbirth were recorded as birth-related trauma. Additionally, the PDS assessed the most upsetting event at T1 DREAM_HAIR_ and whether this event fulfilled the DSM-IV criterion A.

SBE was measured at T2 DREAM by one question: “How was your overall experience of the birth?”. Participants responded on a numeric scale ranging from 0 (*positive*) to 10 (*negative*), which was recoded so higher values represent more positive SBE. This measure has previously shown high predictive value for CB-PTSS (*r* = .34–.39; [9, 58]).

We also included FOC and depressive symptoms during pregnancy as confounding variables. FOC was measured by the Fear of Birth Scale at T1 DREAM (FOBS; [59]). The FOBS economically asks: “How do you feel right now about the approaching birth?” and participants rate on a scale from 0 to 100 how calm (*calm* to *worried*) and fearful (*no fear* to *strong fear*) they felt and answers are averaged. Prenatal depressive symptoms were assessed by the Edinburgh Postnatal Depression Scale (EPDS–German version; [60, 61]) at T1 DREAM. The EPDS asks about the severity of ten symptoms over the past week with four response categories ranging from 0 to 3 (total score: 0 to 30). A cut-off of ≥10 for probable minor and ≥13 for major depression was used [60].

#### Endocannabinoid analyses

Two hair strands (minimum length 2 cm; collective diameter 3 mm) were collected and cut scalp-near from a posterior vertex position. Hair samples were stored at room temperature in aluminium foil in dry and dark surroundings until sent to the laboratory at TU Dresden, Germany, in four batches. Liquid chromatography tandem mass spectrometry was performed according to a validated protocol [38] to quantify hair AEA, 1-AG/2-AG, SEA, PEA, and OEA in the 2 cm hair segment closest to the scalp. Based on an average growth rate of 1 cm per month, this is assumed to reflect endocannabinoid secretion of two months before hair sampling, i.e., approximately the third pregnancy trimester [46].

During endocannabinoid extraction, acyl group migration causes 2-AG to isomerise easily to 1-arachidonoyl-*sn*-glycerol (1-AG), hampering precise determination of 2-AG concentration. As it is assumed that 1-AG originates primarily from 2-AG, the summation of 2-AG and 1-AG levels was used as one variable [38, 62]. Further, due to high intercorrelations between SEA, PEA, and OEA (all *r* = .65–.90, all *p* < .001), scores were averaged and summarised in one variable of N-acyl-ethanolamides (NAE).

### Statistical analyses

Analyses were performed with SPSS 28 [63]. Mean replacement with individuals’ means for the EPDS (*n* = 2) and IES-R (*n* = 10) was used if less than 20% of items were missing. As expected, HEC values were not normally distributed and hence were log-transformed to reduce positive skew. As is common in hair biomarker research [38], outliers ± 3 *SD* from the mean for log-transformed HECs were removed (AEA: *n* = 1; 1-AG/2-AG: *n* = 2; NAE *n* = 2) and non-detectable values were imputed with the lowest measurable score in the sample (AEA: *n* = 19, 7.2%).

Due to residuals showing non-normal distributions, bootstrapping procedures were used. Bias-corrected and accelerated (Bca) bootstrap 95% confidence intervals (CIs) were employed to determine significance. Bonferroni correction was used for multiple regression analyses to correct for multiple testing, resulting in a significant *p*-value of 0.017. Bootstrap resampling procedures were run for regression (2000) and mediation and moderation analyses (5000). Spearman rank-order correlation coefficients were calculated firstly to identify significant confounding variables on HEC (i.e., AEA, 1-AG/2-AG and NAE) to be included in analyses with HEC as the dependent variable and secondly to test correlations between primary study variables. Then, multiple regression analyses were conducted to examine relationships between lifetime trauma and HEC, lifetime trauma and CB-PTSS, and HEC and CB-PTSS. Mediation and moderated mediation analyses were conducted using the ordinary least squares regression-based path analysis modelling tool PROCESS in SPSS [64]. Post-hoc power analysis was run with the software package G*Power [65]. With a sample size of *N* = 263 and an alpha-level of *p* < .05 the statistical power for this study was 0.62 for detecting a small effect (*f*^*2*^ = 0.02) of a single regression coefficient, whereas power exceeded 0.99 for detection of moderate (*f*^*2*^ = 0.15) to large (*f*^*2*^ = 0.35) effect sizes [66].

Based on theoretical deliberations implying an influence of maternal age (e.g., [67, 68]), parity (e.g., [69]), academic degree (e.g., [70, 71]), FOC, and depressive symptoms (e.g., [4, 31]) on lifetime trauma, stress-related hair biomarkers, and CB-PTSS, these were controlled for as confounding variables [72]. Exposure to the COVID-19 pandemic did not affect CB-PTSS (*r*_*s*_ = −.02, *p* = .701), and was therefore not included in analyses with CB-PTSS as the dependent variable. As literature on confounding variables of HEC is scarce, we tested variables that (a) associated with HEC in the only systematic investigation for HEC available (i.e., age, BMI, weekly hair washes; [68]), and (b) have been identified for other hair biomarkers such as cortisol (i.e., hair treatment, hair sample storage time, weekly sun exposure, batch effects, COVID-19 pandemic exposure, and time between hair sampling and birth date as an indicator of gestational age; [46, 49, 73]). The following variables were significantly associated: batch effects with all HEC, COVID-19 pandemic exposure and storage time with AEA and NAE, hair treatment with NAE, and weekly hair washes, weekly sun exposure, BMI, and gestational age at hair sampling with AEA (all *p’s* < .05) (see Supplement 1).

While several outliers were identified showing standardised deleted residuals above 3 [74], closer inspection led to the exclusion of one of them showing the greatest influence and above average HEC values likely due to hair tinting two weeks prior to hair sampling [43, 49, 68]. Analyses are reported excluding this outlier and we report if results changed significantly when including this outlier.

## Results

### Sample characteristics and preparatory analyses

Sample characteristics (*N* = 263) are summarised in Table 1. Participants’ education level was above average for Germany and Dresden with 67.0% reporting an academic degree [75]. The majority was primiparous, and 18 women (6.9%) were likely to suffer from prenatal major depressive disorder. Almost every second mother had experienced at least one potentially traumatic event and almost a third a traumatic event according to DSM-IV (29.8%). CB-PTSS were low on average with four women (1.5%) having an IES-R score above the cut-off.

**Table 1:** Sociodemographic, clinical, and hair-related characteristics (*N* = 263^a^).

Sample characteristics	*n*^a^ (%) or Mean ± *SD* (*Range*) or Mean ± *SD*, Median (*Range*)
Sociodemographic variables^b^	
Age in years (*M, SD, Range*)	30.40 ± 3.93 (18–42)
Mother tongue German (*n*, %)	245 (93.2)
Academic degree (*n*, %)	175 (67.0)
Never married (*n*, %)	134 (51.1)
Primiparous (*n*, %)	217 (82.5)
Psychological variables	
At least one DSM-IV traumatic event (*n*, %)	75 (29.8)
Lifetime trauma (i.e., number of prior potentially traumatic events; PDS)^c^ (*M, SD, Range*)^c^	0.80 ± 1.04 (0–5)
Trauma history^c^	
No Trauma (*n*, %)	135 (51.5)
Accident (*n*, %)	32 (12.2)
Natural disaster (*n*, %)	19 (7.3)
Violent attack (*n*, %)	14 (5.3)
Sexual assault (*n*, %)	22 (8.4)
War, captivity, torture (*n*, %)	0 (0.0)
Illness (*n*, %)	7 (2.7)
Birth-related trauma (*n*, %)	7 (2.7)
Other (*n*, %)	26 (9.9)
Childbirth-related posttraumatic stress symptoms (IES-R total)^d,1^ (*M, SD, Range*)	13.85 ± 10.07 (0–59)
Depressive symptoms (EPDS)^b,2^ (*M, SD, Range*)	5.36 ± 4.04 (0–19)
Fear of childbirth (FOBS)^b,3^ (*M, SD, Range*)	35.19 ± 22.84 (0–100)
Subjective birth experience (SBE)^d^ (*M, SD, Range*)	7.37 ± 2.56 (0–10)
Hair-related variables^c^	
Hair washes per week (*M, SD, Range*)	2.93 ± 1.27 (0.25–7)
Hair treatment (*n*, %)	71 (27.1)
Body mass index (kg/m^2^; *M, SD, Range*)	23.26 ± 3.68 (16.6–40.9)
Storage time in weeks (*M, SD, Range*)	55.82 ± 14.14 (25–85)
Endocannabinoid raw data (pg/mg)^c^	
AEA (*M, SD, Mdn, Range*)	1.14 ± 1.27, 0.79 (0.08–14.59)
1-AG/2-AG (*M, SD, Mdn, Range*)	41.03 ± 29.93, 33.07 (6.89–231.34)
SEA (*M, SD, Mdn, Range*)	968.60 ± 848.21, 728.65 (139.87–5560.12)
PEA (*M, SD, Mdn, Range*)	3471.36 ± 4066.93, 1997.54 (662.58–26592.30)
OEA (*M, SD, Mdn, Range*)	3045.72 ± 4393.86, 1557.13 (177.09–29349.43)
NAE (*M, SD, Mdn, Range*)	2463.79 ± 2963.02, 1513.53 (417.51–19993.51)

*M* mean, *SD* standard deviation, *Mdn* median, *FOBS* Fear Of Birth Scale, *PDS* Trauma list of the Posttraumatic Diagnostic Scale, *IES-R* Impact of Event Scale-Revised, *EPDS* Edinburgh Postnatal Depression Scale, *AEA* anandamide, *1-AG/2-AG* 1- and 2-arachidonoylglycerol, *SEA* stearoylethanolamide, *PEA* palmitoylethanolamide, *OEA* oleoylethanolamide, *NAE* N-acyl-ethanolamides (summed average of SEA, PEA and OEA).

^a^Total *n* varies slightly across variables due to missing values and outlier removal.

^b^T1 DREAM (during pregnancy).

^c^T1 DREAM_HAIR_ (4 ± 2 weeks prior to anticipated birth date).

^d^T2 DREAM (8 weeks after birth).

^1^Cronbach’s α = .79. ^2^Cronbach’s α = .84. ^3^Cronbach’s α = .89.

**Fig. 3:**
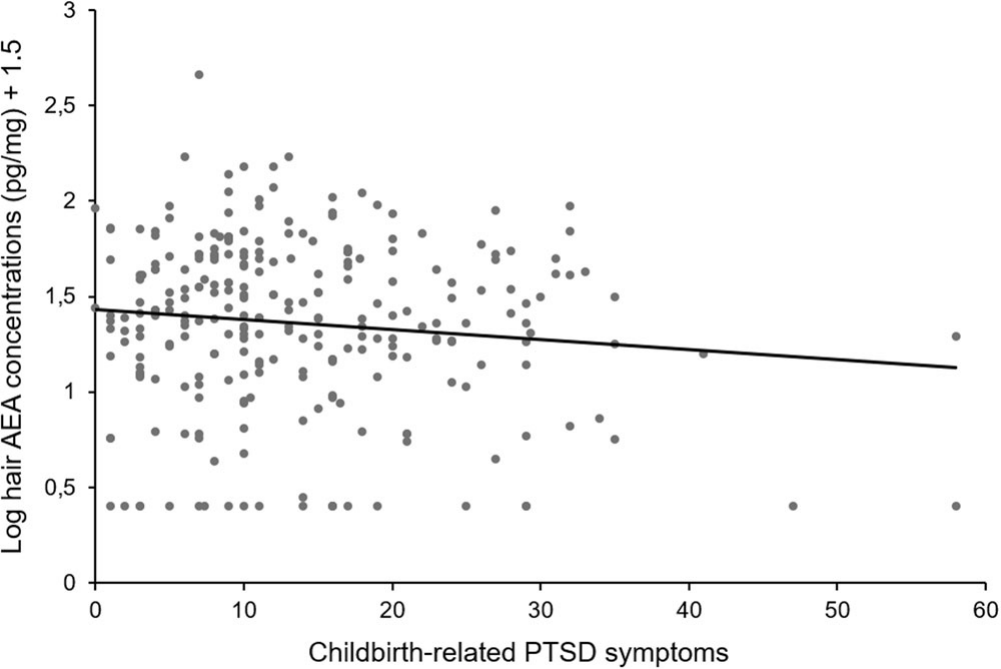
A scatterplot of the relationship between hair AEA and CB-PTSD symptoms with a linear trendline (*N* = 263). *Log-transformed hair AEA levels were used and a constant of* + *1.5 was added for illustrative purposes*.

Correlations between key study variables (Table 2) revealed small to moderate positive associations between endocannabinoids and NAE levels (*r*_*s*_*’s* > .16, *p’s* < .05), however these were not associated with lifetime trauma. CB-PTSS correlated positively with lifetime trauma, FOC, depressive symptoms, and negatively with SBE. CB-PTSS did not correlate significantly with AEA levels (*r*_*s*_ = −.06, *p* = .34, see Fig. 3 for illustration), 1-AG/2-AG levels (*r*_*s*_ = .02, *p* = .70), and NAE levels (*r*_*s*_ = .01, *p* = .93).

**Table 2:** Spearman correlations [95% CI^a^] among primary study variables (*N* = 263^b^).

Variable	1	2	3	4	5	6	7	8
1. CB-PTSS (IES-R)	−	.16^*^ [.04, .28]	−.16^*^ [−.28, −.03]	.20^*^ [.08, .32]	.13^*^ [.01, .25]	−.06 [−.18, .07]	.02 [−.10, .15]	.01 [−.12, .13]
2. Lifetime trauma (PDS)		−	−.08 [−.20, .05]	.17^*^ [.05, .29]	.07 [−.05, .20]	.05 [−.07, .17]	.05 [−.07, .18]	−.01 [−.13, .12]
3. Subjective birth experience			−	−.16^*^ [−.28, −.04]	−.20^*^ [−.32, −.07]	−.02 [−.15, .10]	−.12 [−.24, .01]	−.02 [−.10, .15]
4. Depressive symptoms (EPDS)				−	.39^*^ [.28, .49]	.06 [−.06, .19]	.02 [−.11, .14]	.001 [−.12, .13]
5. Fear of Childbirth (FOBS)					−	.02 [−.11, .14]	.01 [−.12, .13]	.02 [−.11, .14]
6. AEA levels						−	.43^*^ [.32, .53]	.16^*^ [.03, .28]
7. 1-AG/2-AG levels							−	.23^*^ [.11, .35]
8. NAE levels								−

AEA, 1-AG/2-AG, and NAE levels were log-transformed.

*PDS* Trauma Checklist of the Posttraumatic Diagnostic Scale, *IES-R* Impact of Event Scale-Revised, *FOBS* Fear of Birth Scale, *EPDS* Edinburgh Postnatal Depression Scale.

*p ≤ 0.05 (two-tailed).

^a^Values in brackets show the 95% confidence interval for each correlation.

^b^*n* varied slightly due to missing data and outlier removal.

### Main analyses

#### Associations between lifetime trauma and HEC levels during pregnancy

Among expectant mothers, multiple linear regression models, controlling for age, parity, academic degree, FOC, depressive symptoms, and endocannabinoid-specific confounders specified above, showed that lifetime trauma did not significantly predict HECs during pregnancy (AEA: β = .04, *B* = 0.02, 95% BCa CI [−0.04; 0.07], *p* = .579; 1-AG/2-AG: β = .01, *B* = 0.01, 95% BCa CI [−0.03; 0.03], *p* = .851; NAE: β = .03, *B* = 0.01, 95% BCa CI [−0.04; 0.05], *p* = .683; see supplements for detailed results).

#### Predictive associations between lifetime trauma and CB-PTSS

Multiple linear regression, controlling for age, parity, academic degree, FOC, and depressive symptoms, confirmed that the number of prior traumatic experiences predicted CB-PTSS (β = .18, B = 1.66, 95% BCa CI [0.60; 2.89], *p* = .003; see supplements for detailed results).

#### Predictive associations between HEC levels and CB-PTSS

Multiple linear regression models, controlling for age, parity, academic degree, FOC, depressive symptoms, and lifetime trauma, showed that hair AEA levels significantly negatively predicted CB-PTSS (β = −.15, *B* = −3.22, 95% BCa CI [−6.21; −0.43], *p* = .031; see Table 3 and Fig. 3). However, this finding did not survive Bonferroni correction with a significant p-value of .017. When including the one outlier, this effect was significant only at trend-level after bootstrapping procedures were applied (β = −.12, *B* = −2.76, 95% BCa CI [-5.73; 0.25], *p* = .067). Hair 1-AG/2-AG levels (β = −.01, *B* = −0.46, 95% BCa CI [−4.90; 3.84], *p* = .830) and hair NAE levels (β = −.01, *B* = −0.38, 95% BCa CI [−3.57; 2.68], *p* = .811) were not significant predictors of CB-PTSS (see supplements for detailed results).

**Table 3:** Multiple regression analysis predicting maternal CB-PTSS symptoms eight weeks postpartum from maternal hair AEA levels reflecting the third pregnancy trimester, controlling for confounding variables (*N* = 255).

Predictor	ß	*B*	[95% BCa CI]	*p*^a^	R^2^ adj.
					.093
Age	−.12	−0.31	[−0.65, 0.02]	.072	
Parity	−.10	−2.62	[−5.46, 0.02]	.085	
Academic degree	.10	1.95	[−0.98, 4.78]	.189	
Depressive symptoms (EPDS)	.13	0.31	[−0.04, 0.66]	.088	
Fear of Childbirth (FOBS)	.13	0.05	[−0.01, 0.12]	.133	
Lifetime Trauma (PDS)	**.17**	**1.62**	**[0.65, 2.71]**	**.004**	
AEA levels	**−.15**	**−3.22**	**[−6.21, −0.43]**	**.031**^b^	

AEA levels were log-transformed. β = Standardised beta coefficient. B = Unstandardised beta. 95% Bca CI = 95% bias-corrected and accelerated bootstrap confidence interval (2000 iterations), R^2^ adj.  = Adjusted coefficient of determination.

*EPDS* Edinburgh Postnatal Depression Scale, *FOBS* Fear of Birth Scale.

Significant associations (*p* ≤ 0.05) are presented in bold.

^a^bootstrapped *p* values are reported.

^b^*p*-value did not remain significant when Bonferroni corrections due to multiple testing were applied.

#### Mediation analyses

As only AEA showed significant associations in regression analyses, only the mediating effect of AEA in the relationship between lifetime trauma and CB-PTSS was tested in PROCESS Model 4 with age, parity, academic degree, FOC, depressive symptoms, and AEA-specific confounders specified above as confounders. The indirect effect of AEA was not significant (completely standardised indirect effect = −0.01, 95% CI [−0.35, 0.18]).

#### SBE as a moderator of the relationship between lifetime trauma, HEC, and CB-PTSS

**Fig. 4:**
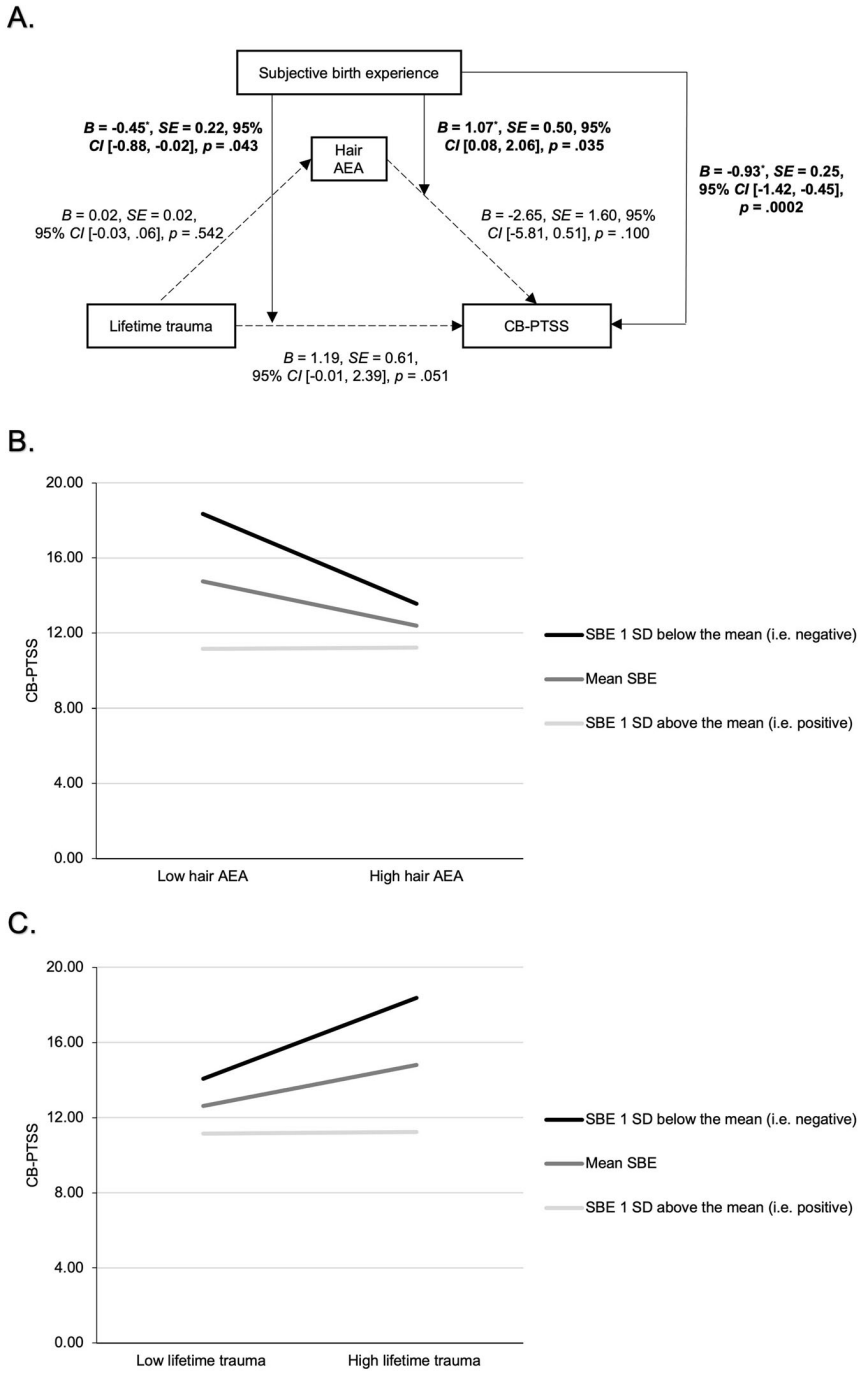
Moderated mediation analysis predicting CB-PTSS. CB-PTSS childbirth-related posttraumatic stress symptoms, AEA anandamide, SBE subjective birth experience. **A** Predicting CB-PTSS based on a moderated mediation analysis with lifetime trauma, hair AEA, and subjective birth experience in PROCESS Model 15. Age, parity, academic degree, FOC, depressive symptoms and AEA-specific confounders were included as confounders. The overall index of moderated mediation was not significant. **B** Graph shows the significant hair AEA x SBE interaction with positive/high and negative/low values corresponding to ± 1 *SD* from the mean. Johnson-Neyman interaction probing showed that reduced hair AEA levels significantly predicted CB-PTSS for SBE values 0.19 *SDs* below the mean and lower (i.e., more negative). **C** Graph shows the significant lifetime trauma x SBE interaction with positive/high and negative/low values corresponding to ± 1 *SD* from the mean. Johnson-Neyman interaction probing showed that the effect of lifetime trauma on CB-PTSS became significant for SBE values 0.01 *SDs* below the mean and lower.

PROCESS Model 15 with mean-centring was employed to examine a moderated mediation model where SBE moderates the relationship between lifetime trauma and CB-PTSS and HEC and CB-PTSS, with age, parity, academic degree, FOC, depressive symptoms, and AEA-specific confounders specified above as confounders. Results showed a nonsignificant overall index of moderated mediation (0.02, 95% CI [−0.05; 0.10]). However, individual interaction effects were significant with SBE moderating both the association between lifetime trauma and CB-PTSS (*B* = −0.45, *SE* = 0.22, 95% CI [−0.88; −0.02], *p* = .042) and the relationship between hair AEA levels and CB-PTSS (*B* = 1.01, *SE* = 0.50, 95% CI [0.08; 2.06], *p* = .035), such that the effect of lifetime trauma and reduced hair AEA on CB-PTSS was greater for mothers with a more negative SBE, compared to those with a more positive SBE (see Fig. 4). When including the one outlier, interactions were no longer significant (*p’s* = .054–.167).

## Discussion

This study aimed to extend aetiological models of CB-PTSS by investigating for the first time the association between maternal HEC during pregnancy and CB-PTSS eight weeks postpartum and their relationship with known risk factors of CB-PTSS. Results build on a small but growing literature examining HEC as a biological risk indicator of stress-related psychopathology (e.g., 39, 40). Findings revealed that before applying multiple-testing corrections reduced maternal hair AEA levels in pregnancy and more lifetime trauma were associated with CB-PTSS and that these predictive effects were stronger upon more negative SBE. No effects were observed for 1-AG/2-AG and NAE.

Consistent with our hypothesis, lower hair AEA concentrations during pregnancy predicted greater CB-PTSS when accounting for relevant confounding variables, however this finding did not survive Bonferroni correction and hence must be interpreted as preliminary. Although this study is the first to investigate the ECS in perinatal mental health and results specifically relate to *CB*-PTSD symptomatology, magnitude and direction of this effect partly align with prior research on *general* PTSD and depressive symptoms. Reduced hair AEA levels were found in borderline personality disorder, often comorbid with PTSD [41], and depression [40], and whilst 2-AG and AEA could not be measured in their study, Wilker et al. [39] found lower hair NAE levels to predict current PTSD symptomatology in war refugees. Yet, our finding contrasts with research amongst emergency medical personnel [43] and refugee minors [44], where hair AEA and PTSS were not associated. The mechanism for a negative relationship between AEA levels and mental health problems remains unclear; however, in line with conceptual models of the ECS, lower AEA levels may reflect impaired regulation of the HPA axis and thereby a greater vulnerability for stress reactions [20]. Such increased vulnerability may, in turn, lead to increased anxiety symptoms, and, as a consequence, increased susceptibility to PTSS after a traumatic event [21, 76], such as a negatively experienced childbirth. We note that the effect of hair AEA changed from trend-level (*p* = 0.067) to actual significance (*p* = .031) only after removal of one outlier and did not survive correction for multiple testing and must therefore be interpreted cautiously and replicated in future research. Taken together, findings tentatively indicate that in a community sample of mothers, lower hair AEA during pregnancy may represent a biological risk indicator for developing CB-PTSS.

While AEA did not mediate the relationship between lifetime trauma and CB-PTSS and overall moderated mediation was not significant, SBE did moderate the relationship of lifetime trauma and hair AEA with CB-PTSS, however only when one outlier was excluded. These findings highlight the importance of the SBE for CB-PTSD development [9], indicating that upon negative SBE, lifetime trauma and hair AEA levels during pregnancy may be more strongly associated with CB-PTSS. This is further consistent with Steudte-Schmiedgen et al.’s. [15] study where lower hair cortisol and cortisone levels during pregnancy predicted CB-PTSS only upon negative SBE. While AEA and cortisol in hair correlated negatively (*r* = −.23, *p* < .001, *n* = 259) in this and another study [40] consistent with conceptual models [20], both AEA and cortisol were also found to be reduced in persons screening positive for depressive disorder [40], suggesting complex time-dependent neuroendocrine processes which require further investigation. Finally, our results underscore the above interpretation that lower AEA levels may reflect physiological vulnerability leading to increased susceptibility for CB-PTSS upon stressor exposure [20] and emphasise a potential buffering effect of positive SBE on the effects of psychological and biological risk factors [77].

Contrary to hypotheses, neither hair 1-AG/2-AG nor NAE levels during pregnancy predicted CB-PTSS. This contrasts with Wilker et al. [39] who found low hair NAE predicted PTSS severity; however, they measured PTSS and HEC *after* the traumatic events had occurred, whereas we measured HEC *prior to* trauma exposure (i.e., childbirth) and assessed PTSS in relation to this event. Hence, NAE levels may change during PTSD yet may not represent a preexisting risk indicator. However, our null finding aligns with previous cross-sectional investigations [43, 44]. One explanation for diverging findings could be that we investigated a very specific population, namely pregnant women, and a specific type of PTSD, namely *CB*-PTSD [78]. When comparing our findings with prior research, these specific conditions need to be considered.

Consistent with the diathesis-stress model of CB-PTSD [4], lifetime trauma, FOC, prenatal depressive symptoms, and SBE significantly correlated with CB-PTSS. In particular the association between lifetime trauma and CB-PTSS aligns with meta-analytic findings indicating trauma history as a risk factor of CB-PTSD [8], and supports the *building block effect* from general PTSD [5] and CB-PTSD [7] literature. Our results highlight that in addition to a dichotomous category of having experienced trauma previously or not, the actual number of prior potentially traumatic experiences may be important for predicting CB-PTSS severity.

Finally, results showed that number of potentially traumatic experiences prior to childbirth were not related to HEC levels during pregnancy. This opposes our hypothesis of a negative association and suggests ECS alterations are not another biological manifestation of the *building block effect* of lifetime trauma similar to cortisol [14, 15]. While research suggests trauma impacts the ECS [21], human research has been heterogeneous regarding direction of effects, finding both negative [39, 42] and positive associations [43]. This heterogeneity could indicate that the effect between trauma and HEC may depend on factors yet to be identified or that they are not causally related. Being the first to examine lifetime trauma and HEC in pregnant women, more research, also in a sample with greater variability in lifetime trauma, is needed. In sum, our results do not support a dose-response relationship in line with a *building block effect* between maternal lifetime trauma and HEC levels during pregnancy.

Core strengths of this study include our large sample of mothers, the prospective design with hair samples taken before birth, long-term endocannabinoid assessment, and assessment of all endocannabinoids and ERCs in hair, particularly AEA, contrasting previous studies (e.g., 42). Moreover, research on CB-PTSD allowed investigation of predictive models as timing of the (potential) trauma (i.e., childbirth) was known [4].

When interpreting findings, it is paramount to consider the study cohort’s unique perinatal state, which is accompanied by pregnancy-specific increases in HEC [46]. Therefore, generalisation to general PTSD literature is limited and should be done carefully. Generalisation is further limited by our sample’s good health and high average education [48]. Less than 2% of participants had CB-PTSS above the clinical cut-off, which is less than the 4.7% suggested by meta-analytic findings [1], which significantly limited the range of CB-PTSS in this study. Hence, the findings only generalise to a subclinically affected sample and future research should aim to compare a clinical and control group. Furthermore, incorporation of endocannabinoids into hair is not fully understood and it remains unclear through exactly which pathways these lipids enter hair and to what extent they reflect endocannabinoid brain activity or plasma endocannabinoid concentrations [79, 80]. Therefore, future research should dedicate itself to these fundamental questions and corroborate our findings to increase confidence in reported effects.

## Conclusion

Findings showed for the first time a role of ECS alterations in postpartum mental health. Our findings complement research into biomarker-based prevention or treatment strategies and may provide a basis for the further development of mechanism-based approaches targeting the ECS in PTSD [81] and CB-PTSD. Specifically, this study showed preliminary results indicating that mothers with reduced hair AEA during pregnancy reported elevated CB-PTSS eight weeks postpartum, while no effects were seen for 1-AG/2-AG and NAE. Furthermore, data revealed that the predictive effect of lifetime trauma and hair AEA for CB-PTSS may be greater for mothers with negative SBE, highlighting the importance of considering subjective peritraumatic experiences. These findings represent an important step in CB-PTSD research and awareness, providing an initial attempt at understanding the biological aetiology of the disorder and paving the way for personalised prevention [3]. Future research may examine pre-pregnancy and perinatal HEC, particularly AEA, as biological risk indicators of CB-PTSS assessed over several assessment points in the postpartum period, accompanied by assessments of traumatic childbirth experiences, also in more clinical samples. Finally, from a transgenerational perspective, it would be important to also examine how offspring outcomes are affected [45]. This as well as the investigation of potentially protective factors, such as social support [82], could valuably inform effective prevention strategies and help identify women at risk for CB-PTSD.

### Supplementary information

Supplementary materials
