# Supplementary material for: Childbirth-related posttraumatic stress symptoms – examining associations with hair endocannabinoid concentrations during pregnancy and lifetime trauma
Source: Transl Psychiatry. 2023 Oct 31;13:335. doi: 10.1038/s41398-023-02610-3 (PMC10618290; doi:10.1038/s41398-023-02610-3)
Supplement: Supplementary file 1 — Supplementary materials [file 41398_2023_2610_MOESM1_ESM.docx]

**Supplementary materials:**

**Childbirth-related posttraumatic stress symptoms – examining associations with hair endocannabinoid concentrations during pregnancy and lifetime trauma**

Luisa Bergunde ^1, 2^, Marlene Karl ^1, 2^, Sarah Schälicke ^1, 2^, Victoria Weise ^1^, Judith T. Mack ^1^, Tilmann von Soest ^3^, Wei Gao ^4^, Kerstin Weidner ^2^, Susan Garthus-Niegel^1, 5, 6 *^, Susann Steudte-Schmiedgen^2 *^

^1^Institute and Policlinic of Occupational and Social Medicine, Faculty of Medicine, Technische Universität Dresden, Dresden, Germany

^2^Department of Psychotherapy and Psychosomatic Medicine, Faculty of Medicine, Technische Universität Dresden, Dresden, Germany

^3^PROMENTA Research Center, Department of Psychology, University of Oslo, Oslo, Norway

^4^Institute of Biological Psychology, Faculty of Psychology, Technische Universität Dresden, Dresden, Germany

^5^Institute for Systems Medicine (ISM), Faculty of Medicine, Medical School Hamburg MSH, Hamburg, Germany

^6^Department of Childhood and Families, Norwegian Institute of Public Health, Oslo, Norway

^*^  shared last authorship

**Supplements 1**

**S1. 1 Results from Spearman correlations between hair-related variables and AEA.**

**Table S1.1**

*Spearman rank-order correlations between hair AEA and potential influential variables (N = 262).*

| **Variable** | **Hair AEA** |
| --- | --- |
| 1. Hair AEA | - |
| 2. Weekly hair washes | ***r* = -.15, *p =* .015** |
| 3. Hair treatment | *r* = .01, *p =* .897 |
| 4. BMI | ***r* = -.14, *p =* .028** |
| 5. Hair sample storage time | ***r* = .28, *p* < .001** |
| 6. Weekly sun exposure | ***r* = -.24, *p* < .001** |
| 7. Gestational age at hair sampling | ***r* = .19, *p =* .002** |
| *Note.* Results in bold are significant at *p* ≤ .05. | |

*Testing for the effects of batch effects and COVID-19 pandemic exposure*

As both the batch effects (four batches, one in 2019, one in 2020, one in 2021, and one in 2022) and exposure to the COVID-19 pandemic are time-based variables, we conducted a hierarchical regression analysis with batch effects in Model 1 (dummy coded with batch 1 as the reference category) and COVID-19 pandemic exposure in Model 2 (see Table S1.2). Batch effect variables were included as confounders in analyses with AEA as the dependent variable if they were significant in Model 1, COVID-19 pandemic exposure was included if it was significant in Model 2.

**Table S1.2**

*Hierarchical regression analysis predicting hair AEA levels.*

| **Variable** | $\boldsymbol{\beta}$ | ***B*** | ***SE*** | ***p*** |
| --- | --- | --- | --- | --- |
| **Model 1** |  |  |  |  |
| Batch 2 vs. Batch 1 | **-.29** | **-0.26** | **0.06** | **<.001** |
| Batch 3 vs. Batch 1 | **.19** | **0.20** | **0.07** | **.004** |
| Batch 4 vs. Batch 1 | **.19** | **0.39** | **0.12** | **.001** |
| **Model 2** |  |  |  |  |
| Batch 2 vs. Batch 1 | **-.29** | **-0.26** | **0.06** | **<.001** |
| Batch 3 vs. Batch 1 | **.38** | **0.41** | **0.09** | **<.001** |
| Batch 4 vs. Batch 1 | **.35** | **0.73** | **0.16** | **<.001** |
| COVID-19 pandemic exposure | **-.29** | **-0.33** | **0.10** | **.001** |
| *Note.* Results in bold are significant at *p* ≤ .05. | | | | |

**S1. 2 Results from Spearman correlations between hair-related variables and 1AG/2AG.**

**Table S1.3**

*Spearman rank-order correlations between hair 1AG/2AG and potential influential variables (N = 261).*

| **Variable** | **Hair 1AG/2AG** |
| --- | --- |
| 1. Hair 1AG/2AG | - |
| 2. Weekly hair washes | *r* = -.07, *p =* .294 |
| 3. Hair treatment | *r* = .02, *p =* .795 |
| 4. BMI | *r* = -.09, *p =* .171 |
| 5. Hair sample storage time | *r* = .05, *p* = .408 |
| 6. Weekly sun exposure | *r* = -.11, *p* = .072 |
| 7. Gestational age at hair sampling | *r* = .08, *p =* .195 |
| *Note.* Results in bold are significant at *p* ≤ .05. | |

*Testing for the effects of batch effects and COVID-19 pandemic exposure*

As both the batch effects (four batches, one in 2019, one in 2020, one in 2021, and one in 2022) and exposure to the COVID-19 pandemic are time-based variables, we conducted a hierarchical regression analysis with batch effects in Model 1 (dummy coded with batch 1 as the reference category) and COVID-19 pandemic exposure in Model 2 (see Table S1.4). Batch effect variables were included as confounders in analyses with 1AG/2AG as the dependent variable if they were significant in Model 1, COVID-19 pandemic exposure was included if it was significant in Model 2.

**Table** **S1.4**

*Hierarchical regression analysis predicting hair 1AG/2AG levels.*

| **Variable** | $\boldsymbol{\beta}$ | ***B*** | ***SE*** | ***p*** |
| --- | --- | --- | --- | --- |
| **Model 1** |  |  |  |  |
| Batch 2 vs. Batch 1 | **-.19** | **-0.11** | **0.04** | **003** |
| Batch 3 vs. Batch 1 | **.36** | **0.25** | **0.04** | **<.001** |
| Batch 4 vs. Batch 1 | **.17** | **0.22** | **0.08** | **.004** |
| **Model 2** |  |  |  |  |
| Batch 2 vs. Batch 1 | **-.19** | **-0.11** | **0.04** | **.003** |
| Batch 3 vs. Batch 1 | **.43** | **0.30** | **0.06** | **<.001** |
| Batch 4 vs. Batch 1 | **.23** | **0.31** | **0.10** | **.003** |
| COVID-19 pandemic exposure | -.11 | -0.08 | 0.07 | .225 |
| *Note.* Results in bold are significant at *p* ≤ .05. | | | | |

**S1. 3 Results from Spearman correlations between hair-related variables and NAE.**

**Table S1.5**

*Spearman rank-order correlations between hair NAE and potential influential variables (N = 261).*

| **Variable** | **1. Hair NAE** |
| --- | --- |
| 1. Hair NAE | - |
| 2. Weekly hair washes | *r* = -.08, *p =* .226 |
| 3. Hair treatment | ***r* = .14, *p =* .024** |
| 4. BMI | *r* = .03, *p =* .607 |
| 5. Hair sample storage time | ***r* = .23, *p* < .001** |
| 6. Weekly sun exposure | *r* = -.06, *p* = .353 |
| 7. Gestational age at hair sampling | *r* = .04, *p =* .564 |
| *Note.* Results in bold are significant at *p* ≤ .05. | |

*Testing for the effects of batch effects and COVID-19 pandemic exposure*

As both the batch effects (four batches, one in 2019, one in 2020, one in 2021, and one in 2022) and exposure to the COVID-19 pandemic are time-based variables, we conducted a hierarchical regression analysis with batch effects in Model 1 (dummy coded with batch 1 as the reference category) and COVID-19 pandemic exposure in Model 2 (see Table S1.6). Batch effect variables were included as confounders in analyses with NAE as the dependent variable if they were significant in Model 1, COVID-19 pandemic exposure was included if it was significant in Model 2.

**Table S1.6**

*Hierarchical regression analysis predicting hair NAE levels.*

| **Variable** | $\boldsymbol{\beta}$ | ***B*** | ***SE*** | ***p*** |
| --- | --- | --- | --- | --- |
| **Model 1** |  |  |  |  |
| Batch 2 vs. Batch 1 | -.02 | -0.01 | 0.05 | .835 |
| Batch 3 vs. Batch 1 | **.15** | **0.12** | **0.06** | **.049** |
| Batch 4 vs. Batch 1 | -.03 | -0.05 | 0.10 | .612 |
| **Model 2** |  |  |  |  |
| Batch 2 vs. Batch 1 | -.02 | -0.01 | 0.05 | .833 |
| Batch 3 vs. Batch 1 | **.31** | **0.25** | **0.08** | **.002** |
| Batch 4 vs. Batch 1 | .11 | 0.17 | 0.14 | .228 |
| COVID-19 pandemic exposure | **-.25** | **-0.22** | **0.09** | **.017** |
| *Note.* Results in bold are significant at *p* ≤ .05. | | | | |

**Supplements 2**

**S2. 1 Results from multiple regression analyses between Lifetime Trauma and HEC.**

**Table S2.1.1**

*Multiple regression analysis predicting hair AEA levels from lifetime trauma (N = 254).*

| **Predictor** |  | ß | *B* | [95% BCa CI] | *p*^a^ | R^2^ adj. |
| --- | --- | --- | --- | --- | --- | --- |
|  |  |  |  |  |  | .313 |
| Age |  | -.03 | -0.00 | [-0.02,0.01] | .670 |  |
| Parity |  | .07 | 0.08 | [-0.05,0.22] | .252 |  |
| Academic degree |  | .08 | 0.07 | [-0.02, 0.16] | .168 |  |
| Hair washes per week |  | -.10 | -0.04 | [-0.07, 0.00] | .069 |  |
| **Storage time** |  | **.27** | **0.01** | **[0.00, 0.01]** | **.003** |  |
| Weekly sun exposure |  | -.01 | -0.00 | [0.00, 0.00] | .829 |  |
| Gestational week at sampling |  | .07 | 0.00 | [-0.00, 0.01] | .249 |  |
| **BMI** |  | **-.13** | **-0.02** | **[-0.03, -0.00]** | **.028** |  |
| Depressive symptoms (EPDS) |  | -.02 | -0.00 | [-0.01, 0.01] | .807 |  |
| Fear of Childbirth (FOBS) |  | -.02 | 0.00 | [-0.00, 0.00] | .751 |  |
| **Batch 2** |  | **-.38** | **-0.34** | **[-0.44, -0.23]** | **.003** |  |
| **Batch 3** |  | **.23** | **0.25** | **[0.11, 0.41]** | **.003** |  |
| **Batch 4** |  | **.24** | **0.49** | **[0.21, 0.82]** | **.003** |  |
| COVID-19 exposure |  | -.17 | -0.20 | [-0.50, 0.08] | .100 |  |
| Lifetime Trauma (PDS) |  | .04 | 0.01 | [-0.04, 0.07] | .579 |  |
| *Note*. AEA levels were log-transformed. β = Standardized beta coefficient. Bca CI = 95% bias corrected and accelerated bootstrap confidence interval (2000 iterations), Adj. *R^2^* = Adjusted coefficient of determination. EPDS = Edinburgh Postnatal Depression Scale. FOBS = Fear of Birth Scale. Significant associations (*p ≤* .05) are presented in bold.  ^a^ bootstrapped *p* values are reported. | | | | | | |

**Table S2.1.2**

*Multiple regression analysis predicting hair 1AG/2AG levels from lifetime trauma (N = 254).*

| **Predictor** |  | ß | *B* | [95% BCa CI] | *p*^a^ | R^2^ adj. |
| --- | --- | --- | --- | --- | --- | --- |
|  |  |  |  |  |  | .288 |
| **Age** |  | **.22** | **0.02** | **[0.01,0.02]** | **<.001** |  |
| Parity |  | -.11 | -0.08 | [-0.17,0.01] | .095 |  |
| Academic degree |  | -.07 | -0.04 | [-0.11, 0.03] | .243 |  |
| Depressive symptoms (EPDS) |  | -.04 | --0.00 | [-0.01, 0.01] | .559 |  |
| Fear of Childbirth (FOBS) |  | -.08 | -0.00 | [-0.00, 0.00] | .216 |  |
| **Batch 2** |  | **-.23** | **-0.13** | **[-0.21, -0.05]** | **.001** |  |
| **Batch 3** |  | **.36** | **0.25** | **[0.16, 0.33]** | **<.001** |  |
| **Batch 4** |  | **.16** | **0.22** | **[0.10, 0.34]** | **<.001** |  |
| Lifetime Trauma (PDS) |  | .01 | 0.01 | [-0.03, 0.03] | .851 |  |
| *Note*. 1AG/2AG levels were log-transformed. β = Standardized beta coefficient. Bca CI = 95% bias corrected and accelerated bootstrap confidence interval (2000 iterations), Adj. *R^2^* = Adjusted coefficient of determination. EPDS = Edinburgh Postnatal Depression Scale. FOBS = Fear of Birth Scale. Significant associations (*p ≤* .05) are presented in bold.  ^a^ bootstrapped *p* values are reported. | | | | | | |

**Table S2.1.3**

*Multiple regression analysis predicting hair NAE levels from lifetime trauma (N = 254).*

| **Predictor** |  | ß | *B* | [95% BCa CI] | *p*^a^ | R^2^ adj. |
| --- | --- | --- | --- | --- | --- | --- |
|  |  |  |  |  |  | .066 |
| Age |  | .13 | 0.01 | [-0.00,0.02] | .080 |  |
| Parity |  | -.00 | -0.00 | [-0.14,0.13] | .950 |  |
| Academic degree |  | -.10 | -0.08 | [-0.18, 0.03] | .134 |  |
| Hair treatment |  | .09 | 0.07 | [-0.02, 0.17] | .131 |  |
| **Storage time** |  | **.17** | **0.00** | **[0.00, 0.01]** | **.006** |  |
| Depressive symptoms (EPDS) |  | -.03 | -0.00 | [-0.02, 0.01] | .710 |  |
| Fear of Childbirth (FOBS) |  | -.04 | -0.00 | [-0.00, 0.00] | .506 |  |
| **Batch 3** |  | **.23** | **0.19** | **[0.08, 0.31]** | **.001** |  |
| COVID-19 exposure |  | -.11 | -0.10 | [-0.22, 0.03] | .153 |  |
| Lifetime Trauma (PDS) |  | .03 | 0.01 | [-0.04, 0.05] | .683 |  |
| *Note*. AEA levels were log-transformed. β = Standardized beta coefficient. Bca CI = 95% bias corrected and accelerated bootstrap confidence interval (2000 iterations), Adj. *R^2^* = Adjusted coefficient of determination. EPDS = Edinburgh Postnatal Depression Scale. FOBS = Fear of Birth Scale. Significant associations (*p ≤* .05) are presented in bold.  ^a^ bootstrapped *p* values are reported. | | | | | | |

**S2. 2 Results from multiple regression analyses between Lifetime Trauma and CB-PTSS.**

**Table S2.2.1**

*Multiple regression analysis predicting CB-PTSS from lifetime trauma (N = 256)*

| **Predictor** |  | ß | *B* | [95% BCa CI] | *p*^a^ | R^2^ adj. |
| --- | --- | --- | --- | --- | --- | --- |
|  |  |  |  |  |  | .075 |
| Age |  | -.10 | -0.25 | [-0.62,0.09] | .153 |  |
| Parity |  | -.11 | -2.78 | [-5.73,0.18] | .066 |  |
| Academic degree |  | .08 | 1.64 | [-1.24, 4.59] | .264 |  |
| Depressive symptoms (EPDS) |  | .12 | 0.28 | [-0.09, 0.62] | .102 |  |
| Fear of Childbirth (FOBS) |  | .13 | 0.06 | [-0.01, 0.13] | .116 |  |
| Lifetime Trauma (PDS) |  | **.18** | **1.66** | **[0.60, 2.89]** | **.003** |  |
| *Note*. β = Standardized beta coefficient. Bca CI = 95% bias corrected and accelerated bootstrap confidence interval (2000 iterations), Adj. *R^2^* = Adjusted coefficient of determination. EPDS = Edinburgh Postnatal Depression Scale. FOBS = Fear of Birth Scale. Significant associations (*p ≤* .05) are presented in bold.  ^a^ bootstrapped *p* values are reported. | | | | | | |

**S2. 3 Results from multiple regression analyses between HEC and CB-PTSS.**

**Table S2.3.1**

*Multiple regression analysis predicting CB-PTSS from 1AG/2AG (N = 254)*

| **Predictor** |  | ß | *B* | [95% BCa CI] | *p*^a^ | R^2^ adj. |
| --- | --- | --- | --- | --- | --- | --- |
|  |  |  |  |  |  | .070 |
| Age |  | -.10 | -0.24 | [-0.61,0.11] | .167 |  |
| Parity |  | -.11 | -2.81 | [-5.76,0.10] | .073 |  |
| Academic degree |  | .08 | 1.70 | [-1.53, 4.76] | .245 |  |
| Depressive symptoms (EPDS) |  | .12 | 0.28 | [-0.05, 0.64] | .112 |  |
| Fear of Childbirth (FOBS) |  | .13 | 0.06 | [-0.01, 0.12] | .124 |  |
| **Lifetime Trauma (PDS)** |  | **.18** | **1.66** | **[0.63, 2.75]** | **.004** |  |
| 1AG/2AG levels |  | -.01 | -0.46 | [-4.90, 3.84] | .830 |  |
| *Note*. 1AG/2AG levels were log-transformed. β = Standardized beta coefficient. Bca CI = 95% bias corrected and accelerated bootstrap confidence interval (2000 iterations), Adj. *R^2^* = Adjusted coefficient of determination. EPDS = Edinburgh Postnatal Depression Scale. FOBS = Fear of Birth Scale. Significant associations (*p ≤* .05) are presented in bold.  ^a^ bootstrapped *p* values are reported. | | | | | | |

**Table S2.3.1**

*Multiple regression analysis predicting CB-PTSS from NAE (N = 254)*

| **Predictor** |  | ß | *B* | [95% BCa CI] | *p*^a^ | R^2^ adj. |
| --- | --- | --- | --- | --- | --- | --- |
|  |  |  |  |  |  | .056 |
| Age |  | -.10 | -0.24 | [-0.57,0.09] | .172 |  |
| Parity |  | -.11 | -2.63 | [-5.61,0.11] | .083 |  |
| Academic degree |  | .07 | 1.39 | [-1.62, 4.26] | .342 |  |
| Depressive symptoms (EPDS) |  | .11 | 0.25 | [-0.08, 0.57] | .134 |  |
| Fear of Childbirth (FOBS) |  | .11 | 0.05 | [-0.01, 0.11] | .180 |  |
| **Lifetime Trauma (PDS)** |  | **.17** | **1.55** | **[0.54, 2.66]** | **.007** |  |
| NAE levels |  | -.01 | -0.38 | [-3.57, 2.68] | .811 |  |
| *Note*. NAE levels were log-transformed. β = Standardized beta coefficient. Bca CI = 95% bias corrected and accelerated bootstrap confidence interval (2000 iterations), Adj. *R^2^* = Adjusted coefficient of determination. EPDS = Edinburgh Postnatal Depression Scale. FOBS = Fear of Birth Scale. Significant associations (*p ≤* .05) are presented in bold.  ^a^ bootstrapped *p* values are reported. | | | | | | |
